# Optimization of ultrasound-assisted extraction based on response surface methodology using HPLC-DAD for the analysis of red clover (*Trifolium pretense* L.) isoflavones and its anti-inflammatory activities on LPS-induced 3D4/2 cell

**DOI:** 10.3389/fvets.2023.1279178

**Published:** 2023-10-02

**Authors:** Zhengqin Luo, Yidan Xu, Longxin Qiu, Shiming Lv, Cheng Zeng, Aijuan Tan, Deyuan Ou, Xuqin Song, Jian Yang

**Affiliations:** ^1^Laboratory of Animal Genetics, Breeding and Reproduction in the Plateau Mountainous Region, Ministry of Education, Guizhou University, Guiyang, Guizhou, China; ^2^Key Laboratory of Preventive Veterinary Medicine and Biotechnology in Fujian Province, Longyan University, Longyan, Fujian, China; ^3^College of Animal Science, Guizhou University, Guiyang, Guizhou, China; ^4^College of Life Science, Guizhou University, Guiyang, Guizhou, China

**Keywords:** red clover, isoflavones, response surface methodology, high-performance liquid chromatography coupled with diode array detection, porcine alveolar macrophage (3D4/2), anti-inflammatory

## Abstract

**Introduction:**

*Trifolium pratense* L. has anti-inflammatory, antioxidant, cardiovascular disease prevention, and estrogen-like effects. The existing method for the assay of effective components is commonly based on a spectrophotometer, which could not meet the requirement of quality control. Furthermore, although there have been many studies on the anti-inflammation effect of red clover, a few have been reported on the regulatory effect of red clover isoflavones (RCI) on lipopolysaccharide (LPS)-induced inflammatory response in porcine alveolar macrophages (3D4/2 cells), and its mechanism of action is still unclear.

**Methods:**

The main components of RCI including daidzein, genistein, and biochanin A were accurately quantified by high-performance liquid chromatography coupled with diode array detection (HPLC-DAD) after optimizing the extraction process through response surface methodology. The anti-inflammatory potential of RCI was carried out by detecting the level of inflammatory cytokines and mRNA expression of related genes. Furthermore, its anti-inflammatory mechanism was explored by investigating two signaling pathways (NF-κB and MAPK).

**Results:**

The optimal extraction conditions of RCI were as follows: the concentration of ethanol is 86% and the solid–liquid ratio is 1:29, with the herb particle size of 40 mesh sieve. Under the optimal conditions, the total extraction of target components of RCI was 2,641.469 μg/g. The RCI could significantly suppress the production and expression of many pro-inflammatory cytokines. The results of the Western blot revealed that RCI dramatically reduced the expression of p65, p-p65, IκB-α, p38, and p-p38. These results are associated with the suppression of the signal pathway of p38 MAPK, and on the contrary, activating the NF-κB pathway. Collectively, our data demonstrated that RCI reversed the transcription of inflammatory factors and inhibited the expression of p65, p-p65, IκB-α, and p38, indicating that RCI had excellent anti-inflammatory properties through disturbing the activation of p38 MAPK and NF-κB pathways.

**Conclusion:**

The extraction conditions of RCI were optimized by HPLC-DAD combined with response surface methodology, which will contribute to the quality control of RCI. RCI had anti-inflammatory effects on the LPS-induced 3D4/2 cells. Its mechanism is to control the activation of NF-κB and p38 MAPK pathways, thereby reducing the expression of inflammatory-related genes and suppressing the release of cytokines.

## 1. Introduction

Inflammation is the body's defense response against the stimulation of various injury factors (such as lipopolysaccharide). Inflammation especially lung inflammation is linked to the occurrence and progression of various animal diseases such as porcine reproductive and respiratory syndrome (PRRS), swine plague, and mycoplasma pneumonia (1), which has become a main threat to pig production. Despite the widespread use of vaccines and antibiotics, lung inflammation in pigs still causes severe economic loss. In addition, controlling the inflammatory response can reduce the damage caused by some pathogens to the body and reduce economic losses (2). It has been shown that LiCl inhibits porcine reproductive and respiratory syndrome virus (PRRSV) infection in PAM-CD 163 cells by enhancing the Wnt/β-catenin pathway and inhibiting the pro-inflammatory response (3). *Crocus sativus* L. protects against sepsis-induced liver, kidney, and lung injury and regulates inflammatory response by inhibiting the p38 MAPK/NF-κB and Bax/Bcl-2 signaling pathways (4). As a significant component of the outer membrane in gram-negative bacteria, lipopolysaccharide (LPS) has become one of the most important stimulators of inflammatory microorganisms (5). It takes on an important responsibility in lung inflammatory injury and can stimulate lung cells to release various pro-inflammatory factors and inflammatory cell factors (6). Porcine alveolar macrophages (PAMs) are a vital part of the body's resistance to pathogenic infection. They not only have the functions of phagocytosis and antigen presentation but also secrete a large amount of biologically active substances, which can enhance the immune inflammatory response and inhibit the occurrence of inflammation. It was previously reported that *H. parasuis* LPS can significantly upregulate the expression of IL-1α, IL-1β, and TNF-α in PAMs when they suffer from stimulation (7). LPS can induce PAMs to produce an inflammatory response and regulate inflammatory reaction through the Notch signaling pathway. Therefore, the study of LPS and PAMs is expected to reflect the pathological mechanism of lung inflammation and provide an important reference for the prevention and control of animal diseases.

Historically, red clover (*Trifolium pratense* L.) has been used locally as an ointment or liquid extract for skin diseases such as psoriasis, eczema, or other rashes, as it is believed that red clover has anti-inflammatory properties. Red clover is a perennial plant of the *Leguminosae* family, which has also become a significant medicinal and useful pasture plant in animal husbandry. It contains many beneficial ingredients such as isoflavones, flavonoids, saponins, and volatile oils. Among them, red clover isoflavone (RCI) is a main and large group of active substances that could exert biological activity. Current studies have isolated and validated multiple isoflavone components from red clover (e.g., biochanin A, daidzein, genistein, irisin, pratensein, and formonp-netin), with the highest contents of biochanin A, daidzein, and genistein (8). Many reports have confirmed that RCI has favorable anti-inflammatory (9) and anti-oxidative effects (10), which exhibit great potential in treating many diseases, such as cancer (11), atherosclerosis (12), osteoporosis (13, 14), and neurodegeneration diseases (15, 16). According to the research, biochanin A can dose-dependently upregulate the expressions of Nrf2 and heme oxygenase 1 in mice suffering from acute liver injury and suppress the synthesis of IL-1β and TNF-α, thereby demonstrating its anti-inflammatory properties (17). In addition, biochanin A can block LPS-induced phosphorylation of RAW264.7 nuclear factor κB (NF-κB) inhibitory protein and p38 mitogen-activated protein kinase (p38 MAPK) and inhibit IL-6, IL-1β, and TNF-α production (18). It has been reported that *Macleaya cordata* extract can inhibit the inflammatory response of PAMs induced by *Salmonella paratyphi* porcine by inhibiting the NF-κB and MAPK signaling pathways (19). Matrine can suppress the production of IL-1β of porcine alveolar macrophages by affecting the MyD 88/NF-κB pathway and NLRP3 inflammasome in the inflammatory response of PRRSV and LPS co-stimulated PAMs (20). Cinnamaldehyde and eugenol inhibit the ability of LPS-induced PAMs to produce TNF-α and could suppress the secretion of IL-1β (21). These results suggested that traditional Chinese medicine can treat the PAM inflammatory response caused by viruses or bacteria. Nevertheless, the function of RCI in PAM inflammation stimulated by LPS, and its mechanism has not been reported yet.

Efficient extraction of natural bioactive compounds is a crucial technological aspect in the deep processing of raw materials, enabling the production of high-value products and maximizing resource utilization. The extraction methods of isoflavones include ultrasonic-assisted extraction, reflux extraction, and water extraction. Ultrasound-assisted extraction is deemed to be the most efficient and fastest way for the extraction of natural active ingredients because it can utilize ultrasonic cavitation, thermal effect, and mechanical effect to destroy the cell wall structure of herbs (22, 23). In addition, ultrasound-assisted extraction facilitates the release of soluble compounds from the plant cells into the solvent, which is time-saving and can increase the extraction efficiency (24). The extraction methods of RCI mainly include organic solvent extraction, microwave-assisted extraction, ultrasonic-assisted extraction, and supercritical CO_2_ extraction, in which organic solvent extraction is the most commonly used method. Generally, the orthogonal experiment is the most commonly used screen optimal extraction condition, while this method can be easily affected by interactive factors, resulting in inaccurate results. More importantly, the traditional strategy for the analysis of isoflavone mainly relies on ultraviolet spectrophotometry, which can not only be quantitated inaccurately but also cannot meet the requirements of quality control. Response surface methodology (RSM), which has the advantages of fewer experimental procedures, low cost, and less time consumption, is widely used to optimize influencing factors in various experiments (25, 26). Moreover, high-performance liquid chromatography (HPLC) provides high sensitivity and unambiguous quantification, which is suitable for the content determination of natural active components and their quality control in herbs. Accordingly, the combination of HPLC and response surface protocol seems as a good alternative for RCI analysis.

To the best of our knowledge, there is no HPLC method combined with response surface methodology for the accurate quantification of the main components in red clover. Although the favorable anti-inflammatory action of RCI is exhibited in the treatment of many other diseases, no study on the anti-inflammation mechanism of RCI in 3D4/2 cells could be acquired. For this purpose, we used response surface methodology to optimize the extraction conditions, and we utilized HPLC-DAD to accurately quantify the main components of red clover (daidzein, genistein, and biochanin A), which will benefit in establishing a quality control system of red clover. Afterward, we established an inflammation model induced by LPS in 3D4/2 cells and evaluated the secretion of inflammatory factors such as IL-1β, IL-6, IL-10, and TNF-α and their mRNA expression levels after RCI treatment. The expression and phosphorylation of proteins (p65, IκB-α, and p38), which are closely related to the NF-κB and MAPK signaling pathways, were investigated. The mechanism of the anti-inflammatory effect of RCI was clarified. This study will help the quality control of red clover and improve the effective utilization of active compounds and also provide a theoretical basis for RCI as a potential product for treating and preventing animal diseases.

## 2. Materials and methods

### 2.1. Chemicals and reagents

Red clover was purchased from Herb Hall (Hebei, China). Dulbecco's Modified Eagle's Medium [DMEM (BM0003)] was acquired from GIBCO BRL Life Technologies (Grand Island, NY, USA). Fetal bovine serum [FBS (11011-8615)] was bought from the products of Zhejiang Tianhang Biotechnology Co., Ltd. (Zhejiang, China). CCK-8 kit (MA0218-2-Sep-08F) was purchased from Meilun Biotechnology Co., Ltd. (Dalian, China). IL-1β (EPC003b), IL-6 (EPC001), IL-10 (EPC010), and TNF-α (EPC002a). ELISA kits were purchased from NeoBioscience Technology Co., Ltd. (Shenzhen, China). Chromatographic grade methanol (B601841) and acetonitrile (B602112) were acquired from Tianjin Kemiou Chemical Reagent Co., Ltd. (Tianjin, China). RIPA lysis buffer (R0010), TRIzol reagent (R1100), BCA kits (PC0020), and ECL Plus (PE0010-A) were purchased from Beijing Solarbio Science & Technology Co., Ltd. (Beijing, China). Antibodies to NF-κB p65 (6,956), p-p65 (3033), MAPK p38 (8,690), p-p38 (4,511), and IκB-α (4,814) were bought from Cell Signaling Technology Co., Ltd. (Danvers, Massachusetts, USA). Antibodies to GAPDH (AF0006) were bought from Beyotime Biotechnology Co., Ltd. (Shanghai, China). All other reagents were of analytical grade.

### 2.2. RCI extraction

Red clover was dried, ground, and passed through the sieve screen. Five gram powder was accurately weighed, and a certain concentration of ethanol was used to extract RCI. Ultrasound-assisted extraction was conducted under different ultrasonic times, temperatures, extraction times, and ethanol concentrations. Then, the extract solution was evaporated using a rotary evaporator and dissolved with 5 ml methanol. Finally, the residue was diluted 10 times and filtered for HPLC analysis.

### 2.3. Determination of three isoflavone contents using HPLC

#### 2.3.1. HPLC conditions

Analyses were achieved using an Agilent Technologies 1260 series HPLC system (Agilent Technologies, Santa Clara, CA, USA) incorporated into a DAD. Sample separation was carried out with an Agilent Extend-C_18_ column (250 × 4.6 mm i.d., 5 μm). The mobile phase consisted of 0.1% formic acid in water solution (A) and acetonitrile (B) with the following gradient elution program: 0–10 min, 30–50% A; 10–15 min, 50–60% A; 15–19 min, 60–50% A; 19–20 min, 50–30% A. The flow rate was set to 1 ml/min with 10 μl of injection volume. The detection wavelength was 254 nm, and the column temperature was 30°C.

#### 2.3.2. Calculation of extraction yield

The contents of daidzein, genistein, and biochanin A in red clover were quantified by external standardization using HPLC. The standard stock solution (2,000 μg/ml) was performed by taking 20 mg of each substance into 10 ml of MeOH. Calibration curves were fitted by analyzing eight standard working solutions (0.5, 1, 5, 10, 15, 30, 50, and 100 μg/ml). The total extraction yield of the main components in red clover isoflavones is calculated by the following formula:


$$\begin{array}{l} x=\frac{\left(m\right)/1000}{M} \end{array}$$

where *x* represents the total extraction yield (μg/g), *m* is the total content of daidzein, genistein, and biochanin A quantified after HPLC determination (μg), and *M* is the weight (g) of dry powder.

### 2.4. Response surface methodology

First, the effects of six factors including water bath temperature, water bath time, ethanol concentration, ultrasonic extraction time, solid–liquid ratio, and medicinal material particle size on the total extraction yield of RCI were evaluated, with the variable design shown in Table 1.

**Table 1:** One-factor experimental design.

**Variables**	**Bath temperature (°C)**	**Water bath time (h)**	**Ethanol concentration (%)**	**Ultrasonic extraction time (min)**	**Solid–liquid ratio (g/mL)**	**Drug particle size (mesh)**
1	20	1	55	0	1:5	10
2	40	2	65	2	1:10	20
3	60	3	75	4	1:15	30
4	80	4	85	6	1:20	40
5	/	5	95	8	1:25	60
6	/	/	/	10	1:30	80
7	/	/	/	/	/	100

To obtain the best extract conditions of RCI, significant variables including ethanol concentration (factor A, %), solid–liquid ratio (factor B, ml/g), and drug particle size (factor C, mesh sieve) were investigated by BOX-Behnken design (BBD) with Design-Expert 13 software. The total extraction yield of RCI is the response-dependent value. Table 2 exhibits the variates and the corresponding levels including predicted and actual values. The second-order polynomial was adopted to fit regression analysis based on the experimental data.

**Table 2:** Factors and levels used in response surface methodology.

**Level**		**Factor**	
	**Solid–liquid ratio**	**Ethanol concentration**	**Drug particle size**
−1	1:20	75%	30 mesh (0.600 mm)
0	1:25	85%	40 mesh (0.425 mm)
1	1:30	95%	100 mesh (0.150 mm)

### 2.5. Cell culture

The 3D4/2 cells were provided by the veterinary pharmacology laboratory of Guangxi University and cultured in DMEM high glucose medium which was supplemented with 12% FBS, 100 EU/ml penicillin, and 100 mg/ml streptomycin at 37°C in a humidified atmosphere containing 5% CO_2_. Cells were passaged after digesting with 0.25% trypsin-EDTA when the cell confluence was between 80 and 90%.

### 2.6. CCK-8 assay for cell viability

The cell viability of RCI to 3D4/2 cells was tested by CCK-8. 3D4/2 cells were cultured in 96-well plates at a density of 5 × 10^4^ cells/ml and allowed to incubate for a period of 12 h. Then, cells were treated with different concentrations of RCI (0, 0.01, 0.05, 0.1, 0.5, 1, 5, and 10 mg/ml) for another 24 h. Afterward, the cells were rinsed twice with phosphate-buffered saline (PBS) and then treated with 90 μl of 2% FBS-DMEM in each well. Subsequently, 10 μl of CCK-8 solution was added. After 1 h of incubation, the absorbance at 450 nm was recorded.

### 2.7. Detection of inflammatory factors such as IL-1β, IL-6, IL-10, and TNF-α

Using 6-well plates, 1 × 10^5^ 3D4/2 cells were seeded in each well and cultured for a whole night. The blank control group was only treated with 2% DMEM. In the experimental groups, 3D4/2 cells were stimulated with LPS (1 μg/ml) for 2 h followed by adding 1 ml RCI (0.25, 0.5, and 1 mg/ml) and dexamethasone (DXMS) to incubate for 24 h. Supernatants were collected, and the release levels of inflammatory factors (IL-1β, IL-6, IL-10, and TNF-α) were measured using the ELISA assay kit.

### 2.8. qRT-PCR analysis

qRT-PCR was used to determine the mRNA level of inflammatory factors mentioned above in the cells. 3D4/2 cells (4 × 10^5^) were challenged with LPS for 2 h followed by incubation with RCI (0.25, 0.5, and 1 mg/ml) and DXMS for 24 h. Total RNA was isolated from the cells using TRIzol reagent, and subsequent cDNA synthesis was performed using a prime script™ RT reagent kit followed by the instructions of the manufacturer. The GAPDH was used as an internal reference. The primer sequences are presented in Supplementary Table S1. The relative gene expression of inflammatory factors was calculated using the 2^−Δ*ΔCt*^ method.

### 2.9. Western blot analysis

The cell treatment method is the same as the step 2.8. After two PBS washes, 300 μl of RIPA lysate buffer containing PMSF and a protease inhibitor was added to the cells, which were then allowed to lyse for 25 min in an ice bath. A BCA protein assay kit was used to determine the protein content. The proteins were transferred to polyvinylidene difluoride membranes after protein separation on 10% SDS-PAGE. After being blocked in 5% non-fat dry milk or BSA for 2 h at room temperature, the membranes were incubated with primary antibodies for a whole night at 4°C. Then, the protein bands were visualized by using the ECL solution after the membranes were incubated with secondary antibodies that were conjugated to HRP.

### 2.10. Statistical analysis

Design-expert 13 and IBM SPSS Statistics 26 data analysis software were used for Box–Behnken experimental design and data analysis. The mean ± standard deviation (x̄ ± SD) was used to represent the data in this research. Single-factor results were compared using the least significant difference method (LSD). The difference between the two groups is considered to be significant when the value is *P* < 0.05, and it is considered highly significant when *P* < 0.01.

## 3. Results

### 3.1. Determination of three isoflavone contents by HPLC

**Figure 1:**
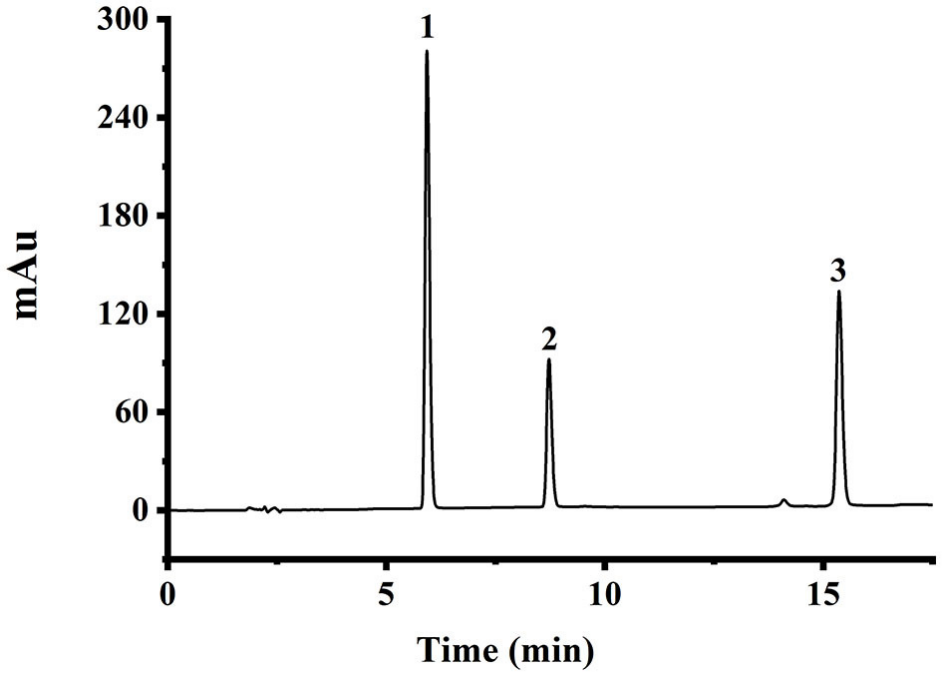
The HPLC chromatogram of mixed standard solution at 10 μg/ml, with peak identification as follows: 1, daidzein; 2, genistein; and 3, biochanin A.

The chromatographic conditions were adjusted slightly based on the study mentioned in Li et al. (27). The HPLC chromatogram of the mixed reference substances under optimal conditions is shown in Figure 1. The separation of chromatographic peaks is good, and the peak shape is sharp, which can meet the requirement of content determination. The calibration curves revealed good linearity within the experimental concentrations of 0.5–100 μg/ml, with their linear regression coefficients (*r*^2^) higher than 0.99 (as shown in Supplementary Table S2).

### 3.2. Optimization of extraction conditions of RCI using response surface methodology

#### 3.2.1. Single-factor experimental analysis

**Figure 2:**
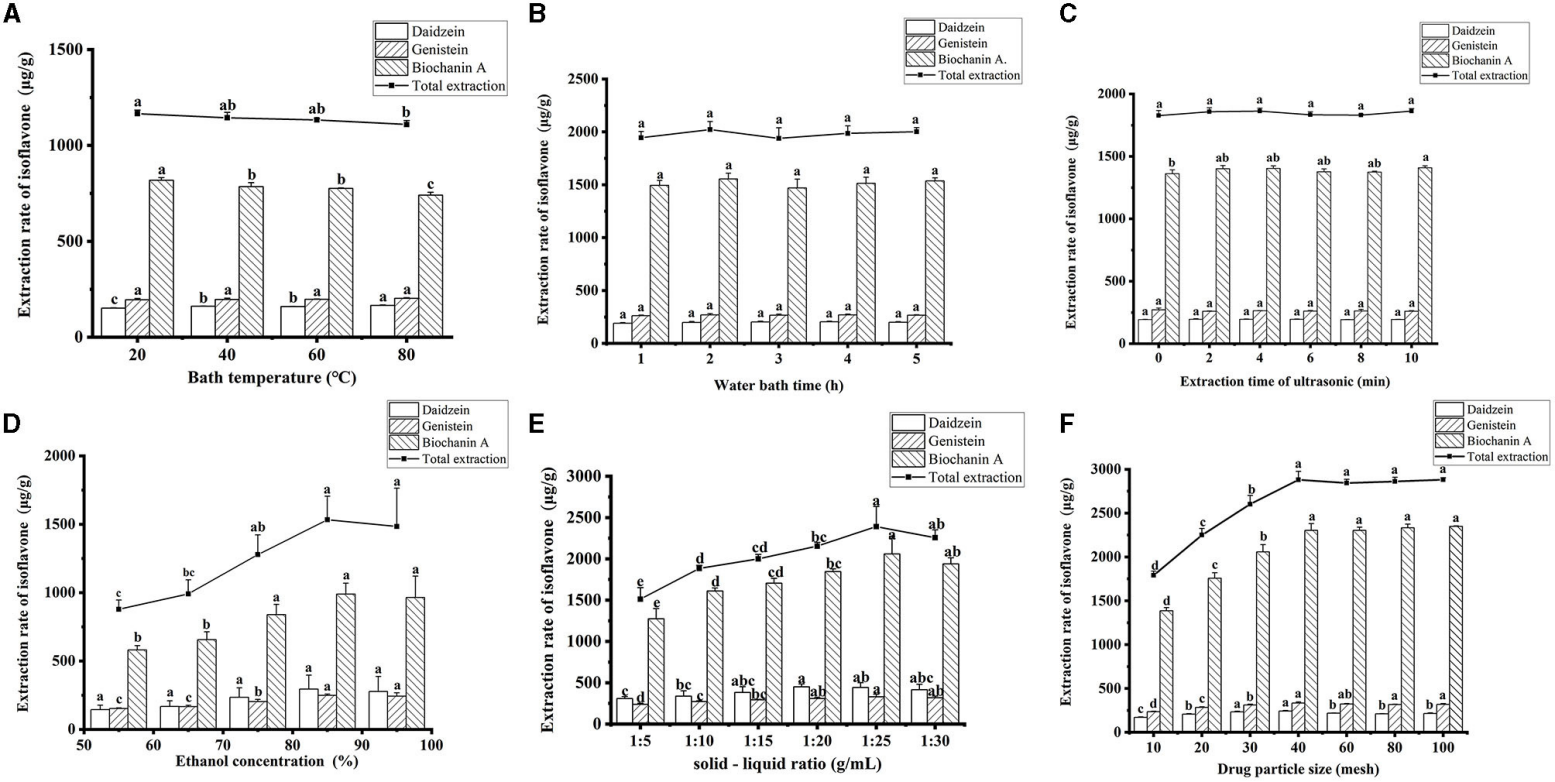
Effects of water bath temperature **(A)**, water bath time **(B)**, ultrasonic time **(C)**, ethanol concentration **(D)**, solid–liquid ratio **(E)**, and drug particle size **(F)** on the total extraction rate of RCI. Values are expressed as mean ± SD for triplicate values. Different lowercase letters indicate that there is a significant difference in the extraction rate of isoflavones between different extraction conditions (*P* < 0.05), and the same lowercase letters indicate that there is no significant difference in the extraction rate of isoflavones between different extraction conditions of RCI (*P* > 0.05).

Main factors affecting the extraction efficiency of RCI, including ethanol concentration, ultrasonic time, solid-liquid ratio, water bath time, bath temperature, and particle size, were optimized. First, single-factor tests were performed at six levels. Figure 2 shows the different factors on the yield of RCI. The water bath time and the ultrasonic time showed no significant impact on both the single component and total extraction rate of RCI (*P* > 0.05). However, the total extraction yield of RCI reached the highest when the water bath time was 2 h and the ultrasonic time was 10 min (Figures 2B, C). Therefore, 2 h of water bath time and 10 min of ultrasonic time were used for further optimization experiments. As for the effect of temperature, the extraction yield of RCI significantly decreased with a rising bath temperature of 20–80°C (*P* < 0.05) (Figure 2A), indicating the poor stability of isoflavones. Considering the extraction yield of RCI and the flexible operation of the water bath, 40°C was selected as the water bath temperature.

The influence of ethanol concentrations on the total extraction yield of RCI was investigated. As shown in Figure 2D, when increasing the ethanol concentration, the total extraction significantly elevated (*P* < 0.05) and reached the highest when the 85% ethanol concentration was used. However, a decrease in RCI yields was observed when the concentration was 95%. In addition, a similar trend of solid–liquid ratios (1:5–1:30 g/ml) was observed (Figure 2E). The yield of RCI improved with increasing the solid–liquid ratio and reached the best when the ratio was 1:25. Generally, decreasing herb particle size can promote extraction efficiency. The influence of different herb particle sizes on the total extraction of RCI is presented in Figure 2F. Particle size has a significant impact on the extraction rate of isoflavones (*P* < 0.05). When the particle size increased from 10 to 40 mesh, the total extraction rate of RCI increased rapidly, and the maximum value appeared at 40 mesh. However, there was no significant change in the extraction rate from 40 to 100 mesh (Figure 2F).

Thus, the optimal conditions for extraction after single-factor analysis were as follows: water bath time, 2 h; ultrasonic time, 10 min; temperature, 40°C; ethanol concentration, 85%; solid–liquid ratio, 1:25; particle size, 40 mesh.

#### 3.2.2. Response surface methodology

**Figure 3:**
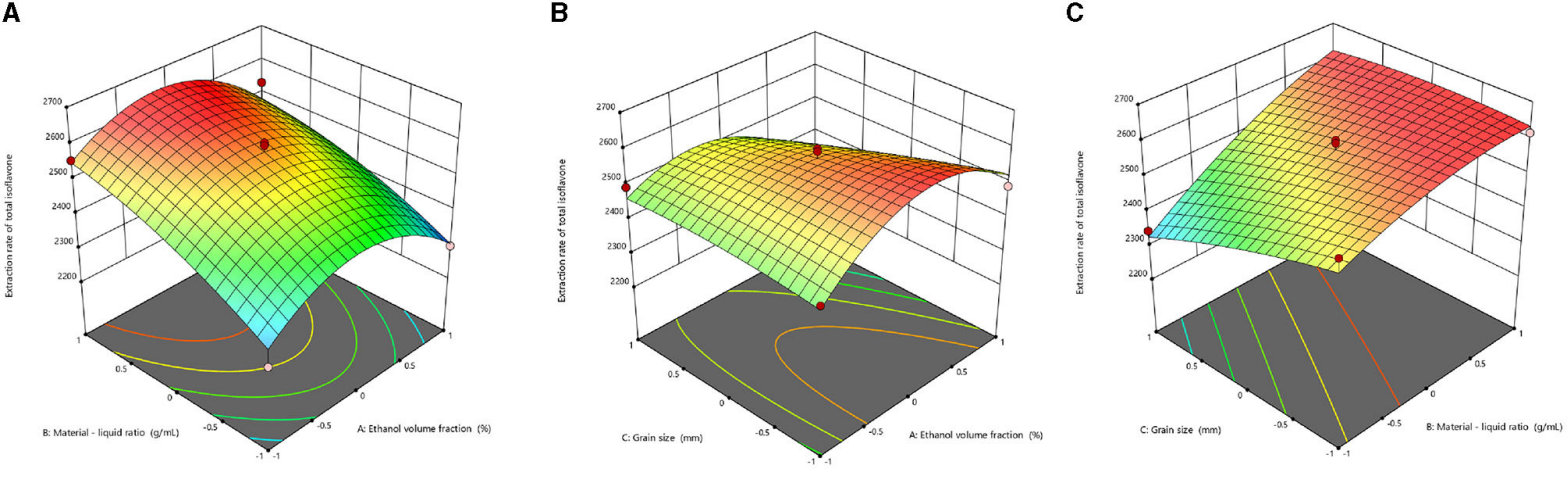
Response surface plot of the effects of solid-liquid ratio and ethanol concentration **(A)**, herb particle size and ethanol concentration **(B)**, solid-liquid ratio and herb particle size **(C)** on the total extraction rate of isoflavones (%).

Design experiments are shown in Supplementary Table S3. Data fitting was based on multiple regression using Design-Expert software, and the regression equation was obtained. The regression equation was as follows:


$$\begin{array}{l} Y=\;2572.635-22.574A+100.306B-53.087C-10.357AB\\ -58.874AC+46.681BC-125.490A^{2}-31.948B^{2}-13.927C^{2} \end{array}$$

where A is the solid–liquid ratio (ml/g), B is the ethanol concentration (%), and C is the drug particle size (mesh sieve). The coefficients of three quadratic terms A^2^, B^2^, and C^2^ are all negative, indicating that there are maximum points. The ANOVA of the response surface model is shown in Table 3. *P*-values were used to investigate the significance level of the model. The results showed that there was a distinct difference in the model with a *P*-value of 0.019 (*P* < 0.05). In addition, lack-of-fit (F-value 2.40, *P* > 0.05) is not significant, implying that the model has a high degree of fitting with the actual situation. The fitting precision of the model was evaluated using the determination coefficients (*R*^2^) and adjusted determination coefficients (*R*^2^ adj). The *R*^2^ was 0.9318 and the *R*^2^ adj was 0.8091, suggesting the favorable fitting precision between the measured and predicted values. The three-dimensional (3D) overall desirability response surface plot for the factors is shown in Figure 3. The slope of the response surface represents the sensitivity of the total yield to the changes in extraction conditions. The contour shape reflects the strength of the interaction, which means the closer to the ellipse, the greater the influence of the interaction. The 3D response surface of ethanol concentration and the solid–liquid ratio are the steepest (Figure 3A), and the contour line is elliptical, indicating the most significant interaction between these two factors. The second significant interaction emerged between the solid–liquid ratio and the particle size (Figure 3B). Furthermore, the interaction between ethanol concentration and particle size was not significant and had the least effect on the total extraction (Figure 3C), which was consistent with the results of ANOVA.

**Table 3:** Variance analysis of regression model.

**Source**	**Sum of squares**	**df**	**Mean square**	***F***	***P***	**Significant**
Model	1.901E+05	9	21,126.97	7.59	0.0190	^*^
A—Ethanol concentration	4,076.61	1	4,076.61	1.46	0.2802	
B—Solid–liquid ratio	80,490.97	1	80,490.97	28.92	0.0030	^**^
C—Grain size	22,546.23	1	22,546.23	8.10	0.0360	^*^
AB	429.03	1	429.03	0.15	0.7108	
AC	13,864.48	1	13,864.48	4.98	0.0760	
BC	8,716.53	1	8,716.53	3.13	0.1370	
A^2^	58,145.81	1	58,145.81	20.89	0.0060	^**^
B^2^	3,768.61	1	3,768.61	1.35	0.2970	
C^2^	716.19	1	716.19	0.26	0.6335	
Residual	13,914.33	5	2,782.87			
Lack of Fit	10,888.05	3	3,629.35	2.40	0.3078	
Pure Error	3,026.29	2	1,513.14			
Cor Total	2.041E+0.5	14				

^*^Represented significant difference (*P* < 0.05).

^**^Represented extremely significant difference (*P* < 0.01).

According to the prediction results, the best conditions for the extraction of RCI were as follows: ethanol concentration of 86.06%, the solid–liquid ratio of 1:29.065 (g/ml), the herb particle size of 0.478 mm, and the predicted maximum extraction yield was 2,630.862 μg/g. Considering the convenience and feasibility in practice, the extraction conditions were adjusted as follows: 86% ethanol aqueous solution and the solid–liquid ratio of 1:29 (g/ml) with a particle size of 40 mesh sieve (0.425 mm). Under these conditions, the extraction was repeated three times, and the actual value (2,641.469 μg/g) was consistent with the predicted value, suggesting that extraction conditions obtained from response surface methodology were highly reliable.

### 3.3. Effects of RCI on the viability of 3D4/2 cells

**Figure 4:**
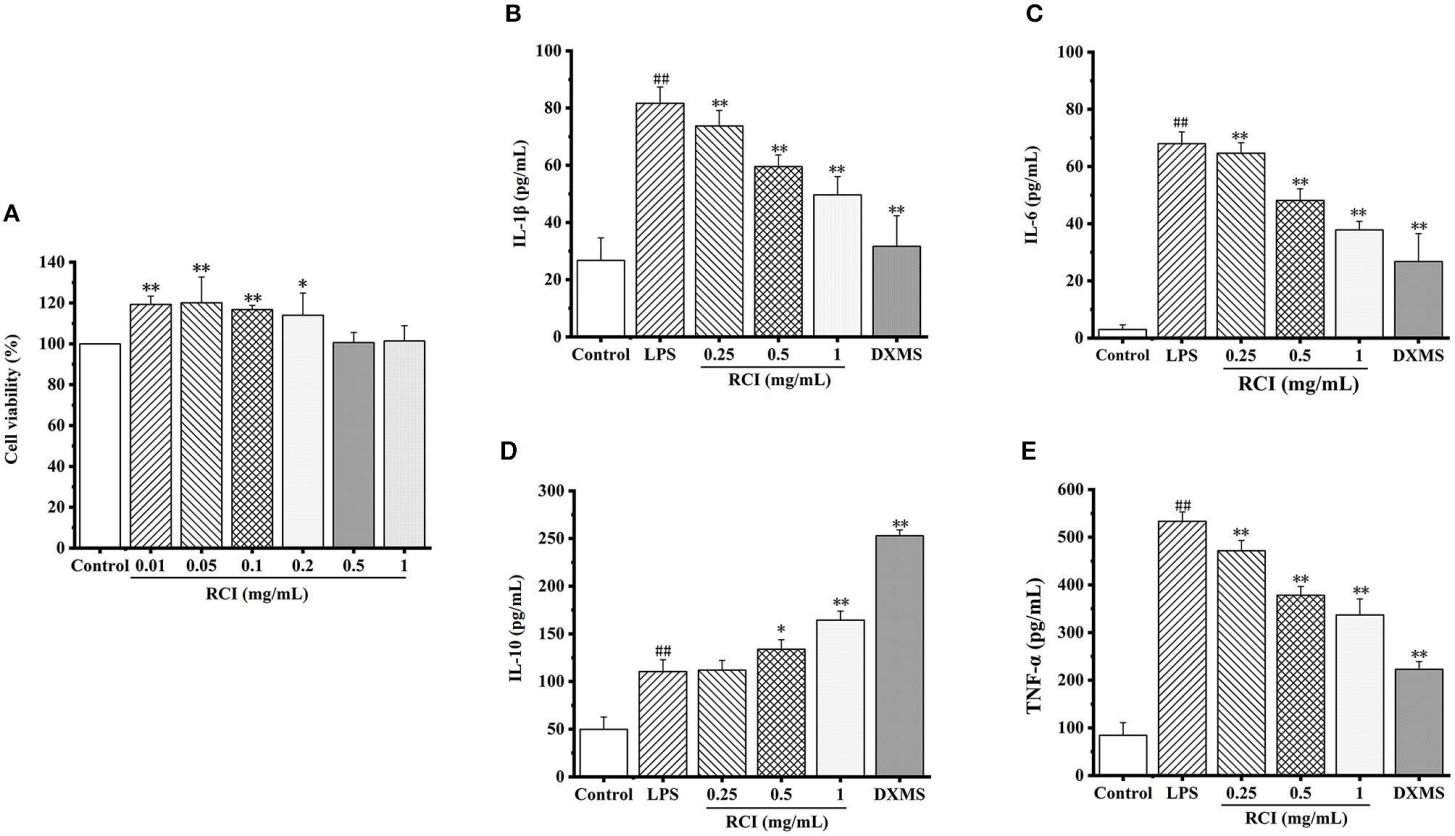
Effect of RCI on the viability of 3D4/2 cells **(A)**. Effect of RCI on IL-1β **(B)**, IL-6 **(C)**, IL-10 **(D)**, and TNF-α **(E)** levels in LPS-induced 3D4/2 cells. Values are expressed as mean ± SD for triplicate values. ^#^*P* < 0.05 and ^##^*P* < 0.01 compared with the control group, *P < 0.05 and **P < 0.01 compared with the LPS alone treated group.

As shown in Figure 4A, 5 mg/ml and 10 mg/m of RCI could decrease cell activity of 3D4/2, and compared with the control group, RCI (0–1 mg/ml) could not inhibit cell viability. Cell viability was higher than 100% at a low concentration of RCI. Therefore, to ensure medical attributes of the drug with no suppressed functions on cell growth, RCI (0.25, 0.5, and 1 mg/ml) was deemed to be the most appropriate concentration.

### 3.4. The effect of RCI on the expression of TNF-α, IL-1β, IL-10, and IL-6 in an *in vitro* LPS-induced inflammation model

The influence of RCI on TNF-α, IL-1β, IL-10, and IL-6 secretion at the dosages of 0.25, 0.5, and 1 mg/ml is shown in Figure 4. In comparison to the control group, LPS increased the content of TNF-α, IL-1β, and IL-6 dramatically, suggesting that the cell inflammation model was established successfully. When different concentrations of RCI were used to deal with cells, RCI could significantly inhibit the secretion of LPS-induced pro-inflammatory cytokines with the dependence of concentration. On the contrary, as an anti-inflammatory cytokine, IL-10 was increased by RCI in a dose-dependent way. These results confirmed that RCI had a positive anti-inflammatory property on LPS-induced 3D4/2 cells.

### 3.5. Effect of RCI on LPS-induced inflammatory cytokines and mRNA expression in 3D4/2 cells

**Figure 5:**
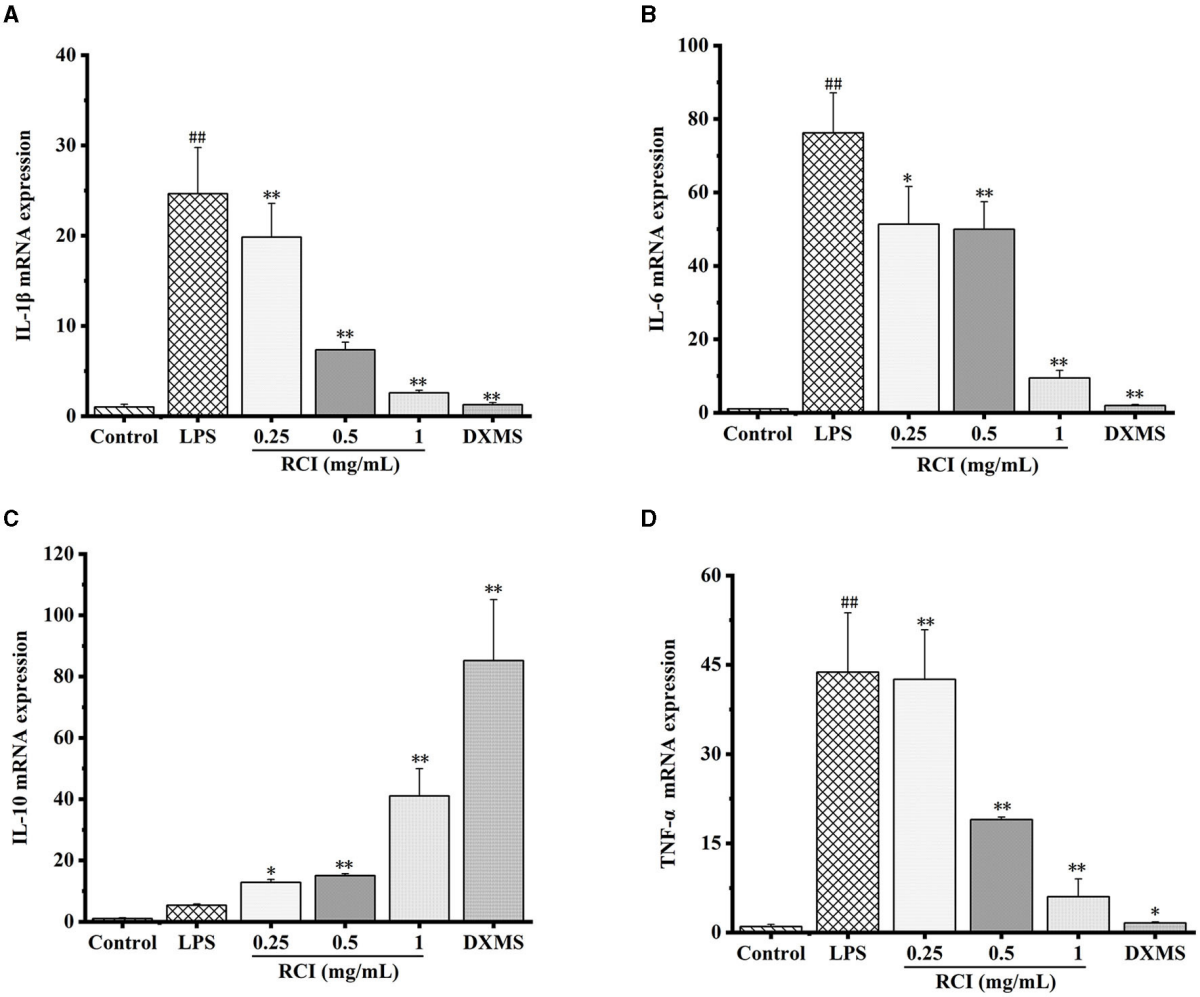
Effect of RCI treatment on the mRNA expression levels of IL-1β **(A)**, IL-6 **(B)**, IL-10 **(C)**, and TNF-α **(D)**. Values are expressed as mean ± SD for triplicate values. ^#^*P* < 0.05 and ^##^*P* < 0.01 compared with the control group, *P < 0.05 and **P < 0.01 compared with the LPS alone treated group.

As presented in Figure 5, 1 μg/ml of LPS could remarkably improve mRNA expression levels of IL-1β, IL-6, and TNF-α (Figures 5A–C). Although the mRNA expression level of IL-10 increased, no significant difference was observed (Figure 5C). Moreover, after samples were co-incubated with RCI, the expression levels of TNF-α, IL-1β, and IL-6 mRNA in 3D4/2 cells decreased in a dose-dependent manner, whereas IL-10 had an opposite trend. Therefore, RCI has a positive effect on combating inflammation by dramatically expressing various inflammatory cytokines at the transcriptional level.

### 3.6. RCI can inhibit the NF-κb and MAPK signaling pathways

**Figure 6:**
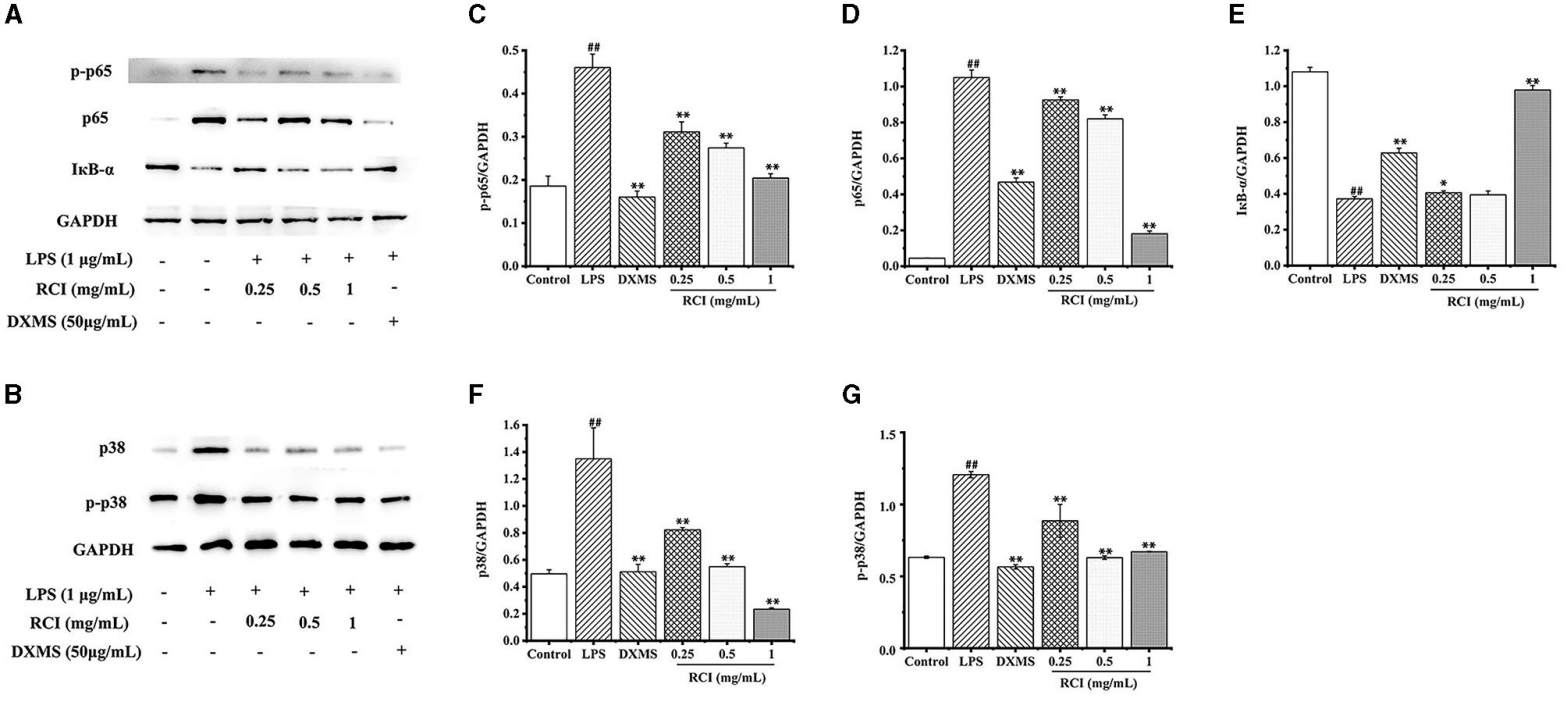
Effect of RCI on LPS-induced protein expression of p65, IκB-α, p38 and p-p38. **(A)** p65 and IκB-α protein expression and phosphorylation determined by western blotting. **(B)** p38 protein expression and phosphorylation determined by western blotting. **(C)** Expression of p-p65 affected by RCI. **(D)** Expression of p65 affected by RCI. **(E)** Expression of IκB-α affected by RCI. **(F)** Expression of p38 affected by RCI. **(G)** Expression of p-p38 affected by RCI. Values are expressed as mean ± SD for triplicate values. ^#^*P* < 0.05 and ^##^*P* < 0.01, compare to control group, *P < 0.05 and **P < 0.01, compare to LPS treated group.

Compared with the control, LPS could obviously enhance the phosphorylation of p65 and p38 and the degradation of IκB-α, and also significantly increase the protein expression of p65 and p38 MAPK. In this study, the RCI at three concentrations of 0.25, 0.5, and 1 mg/ml obviously inhibited the phosphorylation of p65 and p38 and the degradation of IκB-α protein, and significantly reduced the protein expression of p65 and p38 MAPK (Figure 6). Therefore, the results indicated that RCI can inhibit inflammation by controlling the activation of the NF-κB and MAPK pathways.

## 4. Discussion

The ultrasonic-assisted extraction method is a classical strategy to extract active ingredients in plants (28). The extraction process is affected by various factors such as solid–liquid ratio, extraction temperature, time, and ultrasonic power. The response surface analysis is commonly used to solve multivariate problems through the analysis of regression equations (29). This method is different from the “orthogonal experimental design” method promoted in the past. The close connection between factors and test results (response values) can be revealed. Therefore, in this study, response surface analysis was applied to investigate the influence of variables on the ultrasonic-assisted extraction of RCI. Traditionally, ultraviolet spectrophotometry is mostly used for the quantification of isoflavones, which is limited by the reference substances and measurement wavelengths, and as a consequence, there are problems such as large differences between groups and inaccurate quantification. The ambiguous qualitative analysis of active ingredients in herbal medicine using traditional approaches can lead to many issues such as shoddy products and unstable therapeutic effects, which could be detrimental to the industrialization process of herbal products. HPLC has the advantages of high efficiency, high speed, and high sensitivity, playing an important role in the accurate characterization and quality control of active ingredients in traditional Chinese medicine (30). Therefore, to improve the accuracy of content analysis and reduce the experiment's number of extractions, HPLC combined with response surface methodology was used to extract three isoflavones (genistein, daidzein, and biochanin A).

The single-factor test results showed that different factors had different effects on the extraction efficiency of RCI. When the temperature of the water bath increased, the total extraction yield of RCI decreased, which may be due to the degradation of biochanin A at high temperatures. Biochanin A has poor thermal stability, and we can obtain a greater extraction rate under low-temperature conditions, which agrees with the findings of the literature, in which the extraction temperature of biochanin A in the *Sandalwood Leaf* was recommended not higher than 60°C (31).

From the 3D response surface diagram, solid–liquid ratio is an important factor affecting the extraction rate of RCI remarkably. A probable explanation was that an increase in the solid–liquid ratio may increase the contact surface between crude drugs and extraction solvents, which will be beneficial to release isoflavones (32). However, when we continued to improve the solid–liquid ratio, an obvious decrease in extraction yield was observed, resulting from the reduction of ultrasonic energy adhered to the unit volume. Compared with the traditional extraction method of red clover isoflavones, the response surface methodology reduces the concentration of ethanol, the extraction temperature, and the degree of pulverization of medicinal materials and, at the same time, controls the number of extractions to one time, which makes the extraction method more economical, reasonable, and practical.

The active ingredients isolated from red clover differ from plant parts. For example, components isolated from the leaves of red clover are biochanin A, daidzein, genistein, *Iris germanica L*, pretense, and prunus isoflavones while in stems are biochanin A, genistein, and daidzein (33). Therefore, daidzein, genistein, and biochanin A are the main isoflavones in red clover with good pharmacological activities, which have been used in anti-inflammatory and antioxidant. Biochanin A has been proven that it can prevent oxidative stress and inflammatory responses in the liver caused by carbon tetrachloride (10). Liu et al. (34) confirmed the anti-inflammatory activity of biochanin A at the genetic level, and biochanin A can protect liver tissue damage induced by LPS/Ga1N by activating the Nrf2 pathway and reducing NLRP3 inflammasome. Furthermore, biochanin A can treat neutrophil inflammation by activating GPR30 and stimulating cAMP-dependent signaling in inflammation resolution (9). Daidzein has been confirmed to have dose-dependent anti-inflammatory activity on LPS-induced RAW.264.7 macrophages, which inhibits the release of NO, IL-6, and TNF-α and declines iNOS and COX-2 levels, which could be explained by the suppression of the ERK/p38 MAPK and NF-κB p65 pathways (35). This study confirmed that genistein dramatically decreased the production of pro-inflammatory factors and cytokines by activating its corresponding genes. Genistein could bind TLR4 on the surface of microglia competing with LPS to restrain the redox-dependent NF-κB signaling pathway, thereby blocking the activation of LPS-induced intracellular inflammatory signaling cascades (36).

PAMs, as indispensable professional phagocytes and antigen-presenting cells in the lung, play an important role in the immune response against pathogenic infection in pigs (37). Many diseases can cause inflammation in PAMs. PAMs are the main target cell of PRRSV. The PRRSV can cause PAMs to significantly secrete various inflammatory cytokines and produce severe inflammatory reactions (38). *Actinobacillus pleuropneumoniae* exotoxin stimulates the release of PAM pro-inflammatory cytokines and causes porcine pleuropneumonia (39). These cause very large economic losses to the pig farming industry and are not conducive to animal welfare. The inflammatory model induced by LPS in macrophages is a classic model (40). LPS can promote inflammatory cells such as macrophages to secrete various cytokines and cause systemic inflammation. Therefore, to study the use of RCI to inhibit the inflammatory response caused by bacterial diseases and improve the possibility of curing diseases, we used LPS-stimulated 3D4/2 cells as the research model of inflammation. When the 3D4/2 cell is induced by LPS, it triggers an inflammatory reaction. As an essential factor for inflammatory response, IL-1β is closely bound up with cellular functions, such as cell multiplication, differentiation, and apoptosis, which contribute to many different autoinflammatory syndromes (41). Except for mediating inflammatory responses, IL-1β can facilitate the generation of IL-6 and IL-8. IL-6 can regulate anti-inflammatory and pro-inflammatory processes bidirectionally (42). IL-10 can control the lipopolysaccharide-mediated secretion of pro-inflammatory cells. TNF can regulate the function of immune cells and induce inflammation by producing IL-1β and IL-6. In addition, the above-mentioned cytokines can inversely activate the NF-κB pathway and again produce abundant inflammatory factors through the pro-inflammatory pathway, resulting in an inflammatory cascade reaction (43). Numerous researchers have confirmed that TNF-α, IL-1β, and IL-6 would greatly increase the probability of inflammatory reactions (44), while such adverse reactions can be alleviated by blocking the expression of TNF-α, IL-1β, and IL-6 (6). In this study, when 3D4/2 cells were stimulated with LPS, the secretion of pro-inflammatory factors IL-1β, IL-6, and TNF-α was promoted obviously (*P* < 0.01), indicating that *in vitro* inflammation model was successfully constructed. When the cell model dealt with various concentrations of RCI, pro-inflammatory factors decreased significantly in a concentration-dependent manner, while IL-10 increased significantly. In addition, RCI decreased the mRNA levels of IL-1β, IL-6, and TNF-α in a dose-dependent manner. All the results revealed that RCI initiated responses against inflammation by controlling the release of the inflammatory factors.

**Figure 7:**
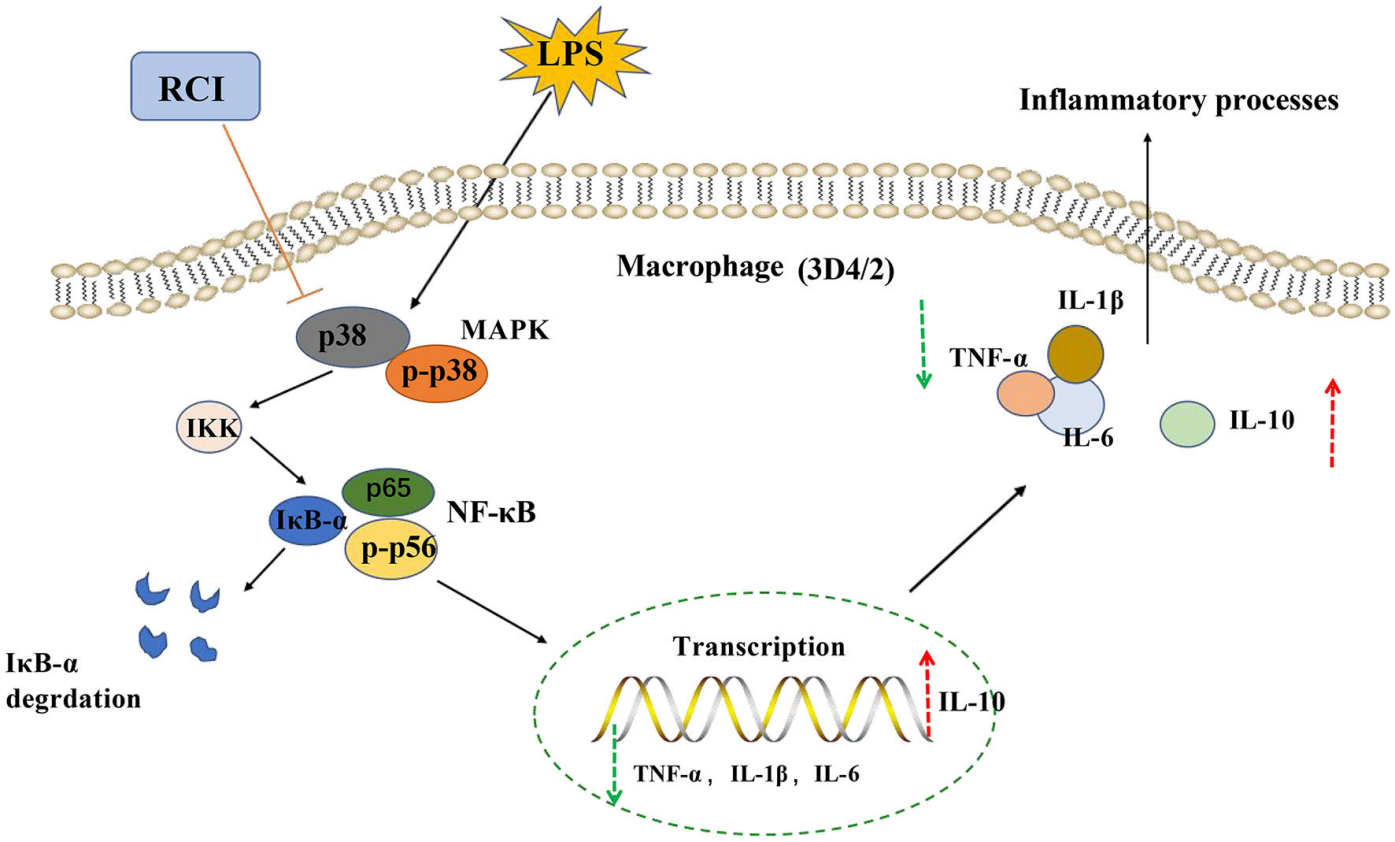
Mechanism diagram of RCI on LPS-induced inflammation signaling pathway in 3D4/2 cells.

NF-κB and MAPK signaling pathways regulate a variety of inflammatory factors, such as IL-12, IL-1β, IL−6, and TNF-α, and inflammatory cell infiltration promotes the occurrence and development of lung tissue inflammation (7). The MAPK signaling pathway includes four subunits, namely, p38 MAPK, c-Jun amino-terminal kinase, ERK, and large mitogen-activated protein kinase (45). p38 MAPK mainly affects the inflammatory process of the body and also participates in cell apoptosis, oxidative stress, and differentiation (46). It has been proven that MAPK is the upstream activator of NF-κB, and p38 MAPK is important in activating and migrating NF-κB to the nucleus (47). After p38 MAPK is activated, it will further activate its downstream NF-κB pathway. NF-κB is a transcription factor that can regulate various reactions such as immune response, inflammation, tumor, cell apoptosis, and cell proliferation. IκB-α degradation Phosphorylation and nuclear translocation of p65 are important events for NFκB signaling activation (4). When cells are induced by LPS, the IκB kinase can be activated, and the downstream IκB protein in the cytoplasm is phosphorylated and rapidly degraded, causing the dissociation of NF-κB p65 and IκB-α and promoting the entry of NF-κB p65 into the nucleus to activate the expression of inflammation-related genes (48). After the p38 MAPK/NF-κB pathway is activated, it can transcribe and induce a variety of inflammatory cytokines, and the downstream inflammatory cytokines gather such as TNF-α, IFN-γ, IL-1β, IL-2, and IL-6, and even the phenomenon of “cytokine storm” is formed, which makes the inflammatory process continue (49). Therefore, the inflammatory response can be inhibited by inhibiting the activation of the NF-κB and MAPK signaling pathways. In this study, Western blotting analysis suggested that RCI significantly inhibited the expression of p65 and p38 protein, inhibited the degradation of IκB-α protein, and weakened the phosphorylation level of P65. The results of ELISA and qRT-PCR also revealed that RCI dose-dependently suppressed the secretion and mRNA expression of inflammatory factors (IL-1β, IL-6, and TNF-α) and promoted the function of IL-10. Therefore, RCI had excellent anti-inflammatory activity on LPS-induced PAMs (3D4/2) and speculated that RCI inhibited the activation of the NF-κB pathway by inhibiting the activation of the p38 MAPK pathway, reducing the secretion of inflammatory cytokines (Figure 7).

## 5. Conclusion

In this study, a sensitive and reliable method using HPLC-DAD combined with the extraction strategy of response surface methodology was developed for the quantitative analysis of RCI. The optimal extraction conditions of RCI were as follows: the concentration of ethanol is 86% and the solid–liquid ratio is 1:29, with the herb particle size of 40 mesh sieve. Under optimal conditions, the extraction yield of target components was 2,641.469 μg/g. In our study, RCI played an anti-inflammatory role by controlling the activation of the NF-κB and MAPK pathways, thereby reducing the expression of related inflammatory genes and the release of cytokines. RCI promises to be an effective drug for porcine inflammatory diseases.

## Data availability statement

The original contributions presented in the study are included in the article/Supplementary material, further inquiries can be directed to the corresponding authors.

## Ethics statement

Ethical approval was not required for the studies on animals in accordance with the local legislation and institutional requirements because only commercially available established cell lines were used.

## Author contributions

ZL: Data curation, Methodology, Writing—original draft, Writing—review and editing. YX: Data curation, Investigation, Methodology, Software, Writing—original draft. LQ: Data curation, Investigation, Writing—review and editing. SL: Data curation, Formal analysis, Methodology, Writing—original draft. CZ: Data curation, Methodology, Writing—review and editing. AT: Data curation, Project administration, Resources, Validation, Writing—original draft. DO: Formal analysis, Investigation, Methodology, Writing—original draft. XS: Formal analysis, Funding acquisition, Writing—original draft, Writing—review and editing. JY: Funding acquisition, Methodology, Project administration, Resources, Supervision, Writing—review and editing, Writing—original draft.
