# Supplementary material for: Optimization of ultrasound-assisted extraction based on response surface methodology using HPLC-DAD for the analysis of red clover (Trifolium pretense L.) isoflavones and its anti-inflammatory activities on LPS-induced 3D4/2 cell
Source: Front Vet Sci. 2023 Oct 2;10:1279178. doi: 10.3389/fvets.2023.1279178 (PMC10580807; doi:10.3389/fvets.2023.1279178)
Supplement: Supplementary file 1 [file Table_1.DOCX]

**Supplementary Information**

**Optimization of ultrasound-assisted extraction based on response surface methodology using HPLC-DAD for the analysis of red clover (*Trifolium pretense* L.) isoflavones and its anti-inflammatory activities on LPS induced 3D4/2 cell**

**Zhengqin Luo ^1,^** **^†^,** **Yidan Xu ^1, †^,** **Longxin Qiu ^2^,** **Shiming Lv ^1, 3^,** **Cheng Zeng ^1, 3^,** **Aijuan Tan ^4^,** **Deyuan Ou ^1,^** **^3^, Xuqin Song ^1, 3, *^ and Jian Yang** **^1, 3,^ ^^[[1]](#footnote-1)^*^**

^1^ Laboratory of Animal Genetics, Breeding and Reproduction in the Plateau Mountainous Region, Ministry of Education, Guizhou University, Guiyang 550025, Guizhou Province, China

^2^ Key Laboratory of Preventive Veterinary Medicine and Biotechnology in Fujian Province, Longyan University, Longyan 364012, Fujian Province, China

^3^ College of Animal Science, Guizhou University, Guiyang 550025, Guizhou Province, China

^4^ College of Life Science, Guizhou University, Guiyang 550025, Guizhou Province, China

| **Table S1** qRT-PCR primer sequences used in this study. | | |
| --- | --- | --- |
| Gene | Forward Primer sequence (5’–3’) | Reverse Primer sequence (5’–3’) |
| IL-1β | AGGGACATGGAGAAGCGATTT | TTCTGCTTGAGAGGTGCTGATG |
| IL-6 | GTCCACTGGGCACATAACT | ACCTGGCTCTGAAACAACA |
| IL-10 | GCATCCACTTCCCAACCA | CTTCCTCATCTTCATCGTCAT |
| TNF-α | TGTAGCCAATGTCAAAGCC | TGCCCAGATTCAGCAAAGT |
| GAPDH | CAAGGAGTAAGAGCCCCTGGA | TGGGATGGAAACTGGAAGTCA |

| **Table S2** Linearity results for each component | | | |
| --- | --- | --- | --- |
| Component | Linear equation | Linear range | *r*^2^ |
| Daidzein | y = 53.576x - 291.22 | 1 ~ 200 | 0.9945 |
| Genistein | y = 64.47x - 668.44 | 1 ~ 200 | 0.9969 |
| Biochanin A | y = 63.601x - 1425.9 | 1 ~ 200 | 0.9906 |

| **Table S3** Design and results of response surface experimental. | | | | |
| --- | --- | --- | --- | --- |
| No | Factor | | | Result of extraction（Y） |
|  | A | B | C | μg/g |
| 1 | -1 | -1 | 0 | 2277.29 |
| 2 | 1 | -1 | 0 | 2296.75 |
| 3 | -1 | 1 | 0 | 2554.36 |
| 4 | 1 | 1 | 0 | 2532.39 |
| 5 | -1 | 0 | -1 | 2461.82 |
| 6 | 1 | 0 | -1 | 2490.53 |
| 7 | -1 | 0 | 1 | 2493.65 |
| 8 | 1 | 0 | 1 | 2286.87 |
| 9 | 0 | -1 | -1 | 2564.22 |
| 10 | 0 | 1 | -1 | 2615.73 |
| 11 | 0 | -1 | 1 | 2344.43 |
| 12 | 0 | 1 | 1 | 2582.66 |
| 13 | 0 | 0 | 0 | 2528.00 |
| 14 | 0 | 0 | 0 | 2599.31 |
| 15 | 0 | 0 | 0 | 2590.59 |

1. * Correspondence author:

   Xuqin Song: song1991yi@163.com

   * Co-Correspondence author:

   Jian Yang, [yangjian0104@163.com](mailto:yangjian0104@163.com)

   **^†^** The authors contributed equally to this work. [↑](#footnote-ref-1)
